# Effect of the Steroid K-canrenoate on *hsp70* Expression and Tissue Damage in Transgenic *Drosophila melanogaster* (*hsp70-lacZ*) *Bg* ^9^

**DOI:** 10.1673/031.012.9201

**Published:** 2012-08-09

**Authors:** Yasir Hasan Siddique, Gulshan Ara, Mohammad Afzal

**Affiliations:** Drosophila Transgenics Laboratory, Section of Genetics, Department of Zoology, Faculty of Life Sciences, Aligarh Muslim University, Aligarh, U.P., 202002, India

**Keywords:** cytotoxicity, heat shock proteins

## Abstract

In the present study the effect of 0.1, 0.2, 0.4, 0.8, and 1.0 µL/mL of the steroid K-canrenoate was evaluated in the third instar larvae of transgenic *Drosophila melanogaster* (*hsp70-lacZ*) Bg^9^ for 6, 24, and 48 hours of duration. The treatment of 0.1, 0.2, and 0.4 µL/mL of K-canrenoate did not induce the activity of *hsp70* significantly compared to the control. The treatments of 0.8 and 1.0 µL/mL of K-canrenoate not only caused tissue damage but also induced a significant increase in the expression of *hsp70* for the different durations of exposure. The results of the present study suggest that the K-canrenoate at 0.8 and 1.0 µL/mL is cytotoxic and caused tissue damage in the third instar larvae of transgenic *D. melanogaster* (*hsp70-lacZ*) Bg^9^.

## Introduction

K-canrenoate is a steroid used in the treatment of hypertension (Martelli et al. 1999). It has been reported to induce single strand breaks in cultured human and rat hepatocytes (Brambilla and Martelli 2002) as well as in rat liver, testes, and ovaries *in vivo* (Martelli et al. 2002). It did not induce single strand breaks in cultured human peripheral blood lymphocytes (Martelli et al. 1999). It induced micronucleus in cultured rat hepatocytes (Brambilla and Martelli 2002) and in rat liver and bone marrow (Martelli et al. 2002), but failed to induce micronucleus in cultured human hepatocytes and peripheral blood lymphocytes (Martelli et al. 1999). It has been reported to increase malignant tumors of the liver, thyroid, brain, and mammary glands in rats at a dose ranging from 20 to 270 mg/kg (Cook et al. 1988).

The use of animals in toxicological research and testing has become an important issue for both science and ethics. As a result, more popular alternatives to mammals have been emphasized in testing, research, and education (Mukhopadhyay et al. 2004), and the European Centre for the Validation of Alternative Methods has recommended the use of *Drosophila* as an alternative model for scientific studies (Festing et al. 1998; Benford et al. 2000). The use of the alternative model *Drosophila* in pharmaceutical research is both time-efficient and cost-effective when compared to rodents. It appears likely that in the future, *Drosophila* will become more widely used to detect adverse drug reactions, and will be helpful in reducing time and cost in the field of drug development (Avanesian et al. 2009).

Genes encoding heat shock proteins (HSPs) are highly conserved, and many of their products can be assigned to families on the basis of sequence homology and molecular weight. In un-stressed cells, HSPs contribute to successful folding, assembly, intracellular localization, secretion, regulation, and degradation of other proteins (Fonager et al. 2002). Under conditions in which protein folding is perturbed or proteins begin to unfold and denature, HSPs assist in protein refolding, protecting cellular systems against protein damages, solubilizing aggregates (to some extent), and sequestering overloaded and damaged protein to degradation machinery (Verbeke et al. 2001; Fonager et al. 2002). All living organisms under stressful conditions respond by synthesizing HSPs (Nover 1991, 1994). HSPs function as molecular chaperones that prevent cellular damage (Bennett and Waters 2000). Elevated temperature is a well-known inducer of heat shock response. However, the role of cold shock is still controversial. The effect of heat shock vs. cold shock has been studied on the expression of *ahsfla*, *zfsf1b*, and *hsp70* in the embryonic cell lines of zebra fish (Airaksinen et al. 2003). Heat exposure increased the ratio of *zhsf1a/b* expression, but cold exposure decreased it to half, suggesting that expression is regulated in a temperature-dependent manner, and supports the use of *hsp70* in stress-response studies (Airaksinen et al. 2003). Other studies also show variation in the heat shock response due to cold and heat exposure; nevertheless, cold exposure remains controversial (Sejerkilde et al. 2003; Bernabo et al. 2011). The expression of hsp70 is detrimental to growth at normal temperatures (Feder et al. 1992; Feder and Krebs, 1998). There is variation in thermotolerance within natural populations of *Drosophila melanogaster* and it is correlated with Hsp70 expression (Krebs and Feder, 1997). The variation in copy number of Hsp70 gene affects Hsp70 concentrations in whole larvae and pupae, which in turn affects their tolerance of natural thermal stress and potentially, their fitness (Feder et al. 1996). In recent years, *hsp70* has been considered to be one of the candidate genes for predicting cytotoxicity against environmental chemicals (Lis et al. 1983; Bierkens 2000; Mukhopadhyay et al. 2002; Mukhopadhyay et al. 2003).

In the present study, the toxicity of K-canrenoate was evaluated by quantifying the *hsp70* expression and tissue damage in the third instar larvae of transgenic *Drosophila melanogaster* Meigen (Diptera: Drosophilidae) (*hsp70-lacZ*) Bg^9^ for different doses and hours of exposure. In addition, this study attempted to determine the level at which no adverse effects of K-canrenoate were observed by stress gene expression in transgenic *D. melanogaster* (*hsp70-lacZ*) Bg^9^.

## Materials and Methods

### Fly strain

A transgenic *D. melanogaster* line that expresses bacterial β-galactosidase as a response to stress was used in the present study (Lis et al. 1983). In this strain of flies, the transformation vector is inserted with a P-element; the line contains wild type *hsp70* sequence up to the *lacZ* fusion point. The flies and larvae were cultured on standard *Drosophila* food containing agar, corn meal, sugar, and yeast at 24 ± 1 ^°^C (Nazir et al. 2003).

### Experimental design

K-canrenoate was dissolved in dimethylsulphoxide, and the 0.1, 0.2, 0.4, 0.8, and 1.0 µL/mL food concentrations were established. The third instar larvae were allowed to feed for different time intervals (6, 24, and 48 hours).

### Soluble O-nitrophenyl-β-D-galactopyranoside (ONPG) assay

The expression of *hsp70* provides a measurement of cytotoxicity (Chowdhuri et al. 1996; Chowdhuri et al. 2001). Methods described by Nazir et al. (2003) were used in this study. After washing in phosphate buffer, larvae were placed in a micro-centrifuge tube (20 larvae/tube, five replicates/group), permeabilized for 10 min by acetone, and incubated overnight at 37 ^°^C in 600 µL of ONPG staining buffer. Following incubation, the reaction was stopped by adding 300 µL of Na_2_CO_3_. The extent of the reaction was quantified by measuring absorbance at 420 nm using Systronics UV/VIS Spectrophotometer 118 (www.systronics.com).

### Trypan blue exclusion test

The extent of tissue damage in larvae caused by the exposure to different concentrations of K-canrenoate was assayed by a dye exclusion test (Nazir et al. 2003; Krebs and Feder 2007). Briefly, the internal tissues of larvae were explanted in a drop of PSS, washed in phosphate buffer saline, rotated in trypan blue stain for 30 min, washed thoroughly in saline, and scored immediately for dark blue staining. About 50 larvae per treatment (10 larvae per dose, five replicates per group) were scored for the trypan blue staining on an average composite index per larva: no color = 0; any blue = 1; darkly stained nuclei = 2; large patches of darkly stained cells = 3; or complete staining of most cells in the tissue = 4 (Krebs and Feder 2009).

### Negative Control

Dimethylsulphoxide at the dose of 1 µL/mL of food acted as a negative control, and the third instar larvae were allowed to feed for different durations.

### Statistical analysis

Statistical analysis was carried out using Student's *t-test* with commercial software Statistica (www.statsoft.com).

## Results

**Figure 1.**
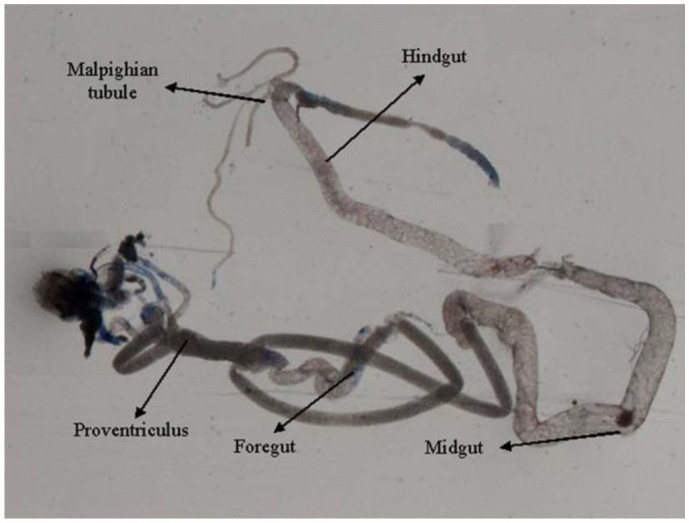
Trypan blue staining pattern in the third instar larval tissues of *Drosophila melanogaster* (*hsp70-lacZ*) Bg^9^ after the exposure of 0.8 µL/mL of potassium canreonate for six hours. High quality figures are available online.

**Figure 2.**
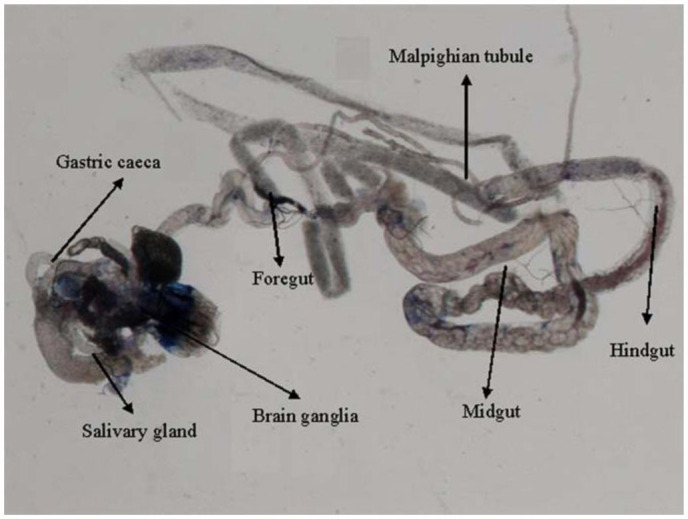
Trypan blue staining pattern in the third instar larval tissues of *Drosophila melanogaster* (*hsp70-lacZ*) Bg^9^ after the exposure of 0.8 µL/mL of potassium canreonate for 24 hours. High quality figures are available online.

**Figure 3.**
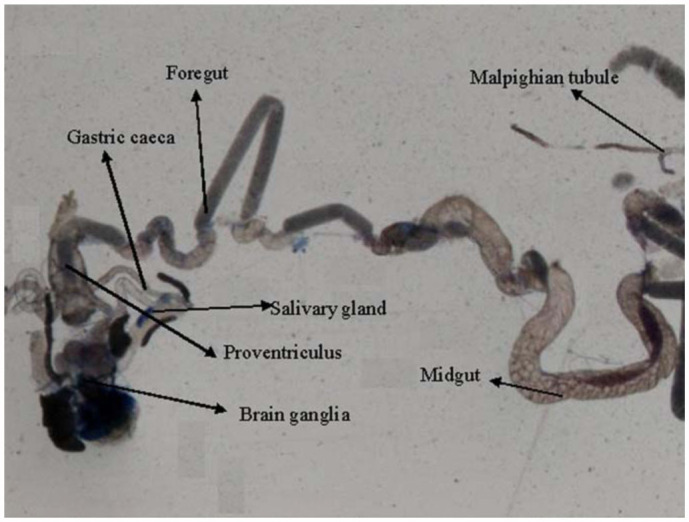
Trypan blue staining pattern in the third instar larval tissues of *Drosophila melanogaster* (*hsp70-lacZ*) Bg^9^ after the exposure of 0.8 µL/mL of potassium canreonate for 48 hours. High quality figures are available online.

**Figure 4.**
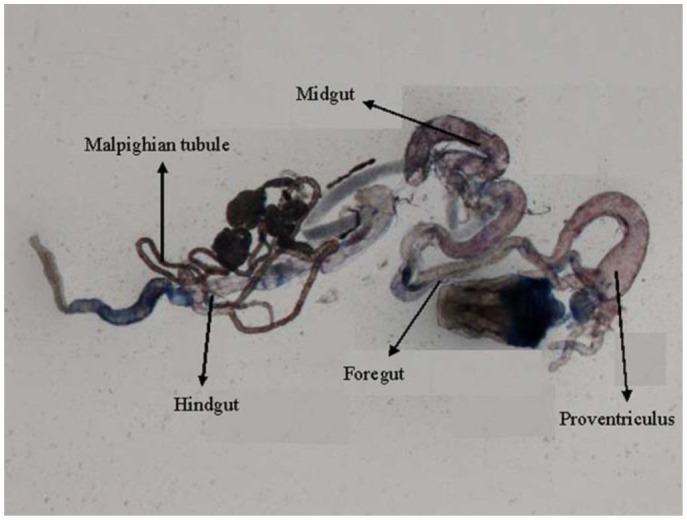
Trypan blue staining pattern in the third instar larval tissues of *Drosophila melanogaster* (*hsp70-lacZ*) Bg^9^ after the exposure of 1.0 µL/mL of potassium canreonate for six hours. High quality figures are available online.

**Figure 5.**
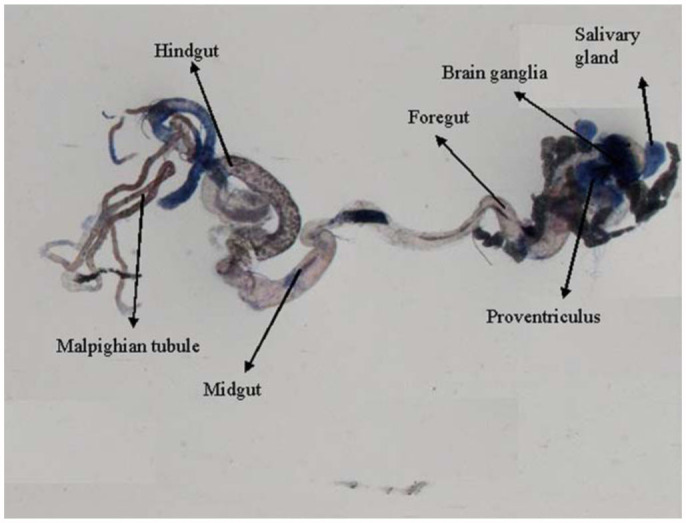
Trypan blue staining pattern in the third instar larval tissues of *Drosophila melanogaster* (*hsp70-lacZ*) Bg^9^ after the exposure of 1.0 µL/mL of potassium canreonate for 24 hours. High quality figures are available online.

**Figure 6.**
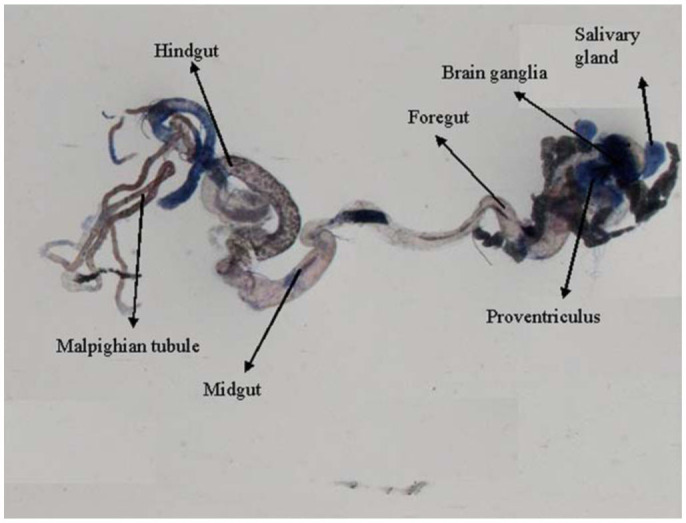
Trypan blue staining pattern in the third instar larval tissues of *Drosophila melanogaster* (*hsp70-lacZ*) Bg^9^ after the exposure of 1.0 µL/mL of potassium canreonate for 48 hours. High quality figures are available online.

The treatment with 0.1, 0.2, and 0.4 µL/mL of K-canrenoate did not result in any significant increase in the β-galactosidase activity for various time intervals, and β-galactosidase activity was equivalent to that observed in the control (Table 1). Treatment with 0.8 µL/mL of K-canrenoate showed an increase in the β-galactosidase activity with an increase in exposure time (Table 1). The increased activity of β-galactosidase at this concentration was significant (*p* < 0.01) when compared to the control. However, at an even higher concentration of K-canrenoate (i.e., 1.0 µL/mL), β-galactosidase activity decreased, but still was significant as compared to the control. The negative control was associated with mean values of 0.2331, 0.2211, and 0.2318 for 6, 24, and 48 hours of exposure, respectively (Table 1). Regression analysis was also performed for the dose, duration, and β-galactosidase activity (Table 2). The treatment of 0.1, 0.2, 0.4, 0.8, and 1.0 µL/mL of K-canrenoate for six hours of exposure was associated with an r value of 0.8990. Similarly, the treatments of 0.1, 0.2, 0.4, 0.8, and 1.0 µL/mL of K-canrenoate for 24 and 48 hours of exposure were associated with r values of 0.66909 and 0.45476, respectively. Trypan blue staining was performed to study whether the K-canrenoate could induce damage in the tissues of exposed larvae. About 90% of the larvae in controls and negative controls were negative for trypan blue staining even after 48 hours of the treatment (Figures not shown). About 85% of larvae exposed to 0.1, 0.2, and 0.4 µL/mL of K-canrenoate were also negative for the trypan blue staining, although the remaining 15% showed a light blue stain in the head region (Figures not shown). About 90% of larvae exposed to 0.8 µL/mL of K-canrenoate for six hours showed staining in the head region, gastric caecae, and a light blue color in the foregut (Figure 1). About 90% of larvae exposed to 0.8 µL/mL of K-canrenoate for 24 hours showed staining in the head region and gastric caecae (Figure 2). About 90% of larvae exposed to 0.8 µL/mL of K-canrenoate for 48 hours show staining in the head region, gastric caecae, and salivary gland, and showed a light blue color in the foregut region (Figure 3). About 95% of larvae exposed to 1.0 µL/mL of K-canrenoate for six hours showed staining in the head region, gastric caecae, salivary gland, and proventriculus, a light blue color in the foregut region, and a very light blue color in the hindgut (Figure 4). About 95% of larvae exposed to 1.0 µL/mL of K-canrenoate for 24 hours showed a staining in the head region, brain ganglia, gastric caecae, salivary gland, and proventriculus, a light blue color in the foregut and midgut regions, and a blue color in the hindgut (Figure 5). About 95% of larvae exposed to 1.0 µL/mL of K-canrenoate for 48 hours showed a staining in the head region, brain ganglia, gastric caecae, salivary gland, proventriculus, and malpighian tubule, a light blue color in the foregut and midgut regions, and a blue color in the hindgut (Figure 6).

**Table 1.**
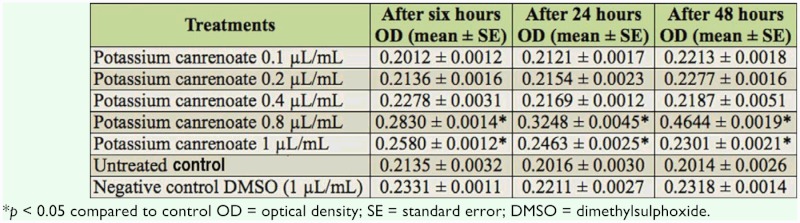
β-galactosidase activity measured in transgenic *Drosophila melanogaster* (*hsp70-lacZ*) Bg^9^ third instar larvae exposed to different concentrations of K-canrenoate for various time intervals.

**Table 2.**
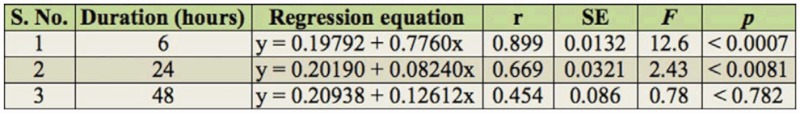
Regression analysis for the β-galactosidase activity in transgenic *Drosophila melanogaster* (*hsp70-lacZ*) Bg^9^ third instar larvae exposed to different concentrations of potassium canreonate for various time intervals.

## Discussion

The results of the present study reveal that K-canrenoate did not to induce *hsp70* significantly (compared to untreated), as evidenced by the β-galactosidase activity in third instar larvae of transgenic *D. melanogaster* (*hsp70-lacZ*) Bg^9^ at 0.1, 0.2, and 0.4 µL/mL of food. The doses of 0.8 and 1.0 µL/mL, however, induced significant expression of *hsp70*. Expression of *hsp70* in the tissues was found to increase for the 0.8 µL/mL of K-canrenoate with an increase in duration. The decrease in activity of the β-galactosidase at 1.0 µL/mL of K-canrenoate for even the six hours of exposure was due to tissue damage at the higher concentration, as also evidenced by the trypan blue staining. However, there are reports of auto repression of *hsp70* once its upper limit of cellular level is reached (Di Domenico et al. 1982; Craig et al. 1989; Stone and Craig 1990; Nover 1994). The possibility of reduction in the number of viable cells after 48 hours of exposure at the highest concentration (1.0 µL/mL) or the instability of the reporter gene after 48 hours at this concentration may also be responsible for the decreased activity of *hsp70* (Mukhopadhyay et al. 2002). Although having protective roles in living systems, HSPs are being exploited by toxicologists (Guven et al. 1994; Guven and Pomerai 1995; Chowdhuri et al. 1996; Chowdhuri et al. 2001; Nazir et al. 2003).

As an effective biosensor to even a minor stimulus, *hsp70* expression is considered to be an effective marker for toxicological evaluations (Mukhopadhyay et al. 2003). The use of animals for toxicological evaluations has become a fundamental concern for scientists, not only for social and ethical reasons, but also because of difficulty in interpreting data due to intra species variation and exorbitant costs (Benford 2000; Mukhopadhyay et al. 2003). This has led researchers to encourage the use of alternative animals in toxicological evaluations (Mukhopadhyay et al. 2003). *Drosophila* is a well-established animal model for geneticists as well as developmental and molecular biologists. In the past years, a significant contribution has been made by successfully employing transgenic *D. melanogaster* as an alternative animal model for toxicological research (Chowdhuri et al. 1996; Chowdhuri et al. 2001; Mukhopadhyay et al. 2002; Mukhopadhyay et al. 2003). An early study with *Drosophila* suggested that the induction of HSPs following cold exposure (0 ^°^C) was due to the shift from 0 ^°^C back to the control temperature of 25 ^°^C, i.e., heat shock, rather than a result of the cold treatment itself (Burton et al. 1988).

In the present study, a clear dose and duration response was observed (above 0.25 µL/mL of diet) on *hsp70* induction. HSPs are formed in response to stressors like LPO, DNA damage, osmotic imbalance, protein misfolding, membrane perturbation, metals, heat shock, etc. (Nazir et al. 2003). A dose-dependent increase in the activity β-galactosidase clearly demonstrates the dose-dependent toxic effects of K-canrenoate in transgenic *D. melanogaster* (*hsp70-lacZ*) *Bg^9^*, and strengthens the utility of *hsp70*_expression as bio-indicator of exposure to environmental chemicals. The metabolic activation of K-canrenoate leads to the formation of 3α and 3β hydroxy derivatives via β-epoxide, which act as mutagens (Cook et al. 1988, 1993; Martelli et al. 1999). Our earlier studies with the steroids in cultured human lymphocytes and in mice also suggest the formation of reactive oxygen species responsible for genotoxic damage (Siddique and Afzal 2004, 2005; Siddique et al. 2005a, 2005b, 2006). The highest concentration of the cytochromes and microsomal oxidase activity has been reported in the midgut of the insects (Wilkinson and Brattesten 1972). Our findings indicate that K-canrenoate is toxic at 0.8 and 1.0 µL/mL only; however, the concentration produced in humans by the therapeutic dose of 200 mg is 3.18 µg/mL (Ramsay et al. 1976). The present study also suggests that K-canrenoate at 0.4 µL/mL has no observed adverse effects on *Drosophila,* and provides evidence of the cytotoxic potential of K-canrenoate at and above 0.8 µL/mL on non-target organisms like *Drosophila.* These results call for further examination of stress gene expression as a potential indicator of non-target toxicity.

## Abbreviations

HSP,: heat shock protein
